# Efficacy of APX2039 in a Rabbit Model of Cryptococcal Meningitis

**DOI:** 10.1128/mbio.02347-22

**Published:** 2022-10-12

**Authors:** Charles D. Giamberardino, Wiley A. Schell, Jennifer L. Tenor, Dena L. Toffaletti, Julia R. Palmucci, Choiselle Marius, Jane-Valeriane K. Boua, Quinlyn Soltow, Robert Mansbach, M. Arthur Moseley, J. Will Thompson, Laura G. Dubois, William Hope, John R. Perfect, Karen Joy Shaw

**Affiliations:** a Duke University School of Medicine, Department of Medicine, Division of Infectious Diseases, Durham, North Carolina, USA; b Duke University School of Medicine, Department of Surgery, Durham, North Carolina, USA; c Pfizer, Inc., La Jolla, California, USA; d Mansbach Consulting, LLC, San Diego, California, USA; e Duke University School of Medicine, Duke Proteomics and Metabolomics Core Facility, Center for Genomic and Computational Biology, Durham, North Carolina, USA; f Antimicrobial Pharmacodynamics and Therapeutics, University of Liverpool, Liverpool Health Partners, Liverpool, United Kingdom; g Hearts Consulting Group, LLC, Poway, California, USA; h Duke University School of Medicine, Department of Neurosurgery, Durham, North Carolina, USA; University of British Columbia

**Keywords:** *Cryptococcus*, APX2039, APX001, fosmanogepix, manogepix, Gwt1, gepix, antifungal, meningitis

## Abstract

Cryptococcal Meningitis (CM) is uniformly fatal if not treated, and treatment options are limited. We previously reported on the activity of APX2096, the prodrug of the novel Gwt1 inhibitor APX2039, in a mouse model of CM. Here, we investigated the efficacy of APX2039 in mouse and rabbit models of CM. In the mouse model, the controls had a mean lung fungal burden of 5.95 log_10_ CFU/g, whereas those in the fluconazole-, amphotericin B-, and APX2039-treated mice were 3.56, 4.59, and 1.50 log_10_ CFU/g, respectively. In the brain, the control mean fungal burden was 7.97 log_10_ CFU/g, while the burdens were 4.64, 7.16, and 1.44 log_10_ CFU/g for treatment with fluconazole, amphotericin B, and APX2039, respectively. In the rabbit model of CM, the oral administration of APX2039 at 50 mg/kg of body weight twice a day (BID) resulted in a rapid decrease in the cerebrospinal fluid (CSF) fungal burden, and the burden was below the limit of detection by day 10 postinfection. The effective fungicidal activity (EFA) was −0.66 log_10_ CFU/mL/day, decreasing from an average of 4.75 log_10_ CFU/mL to 0 CFU/mL, over 8 days of therapy, comparing favorably with good clinical outcomes in humans associated with reductions of the CSF fungal burden of −0.4 log_10_ CFU/mL/day, and, remarkably, 2-fold the EFA of amphotericin B deoxycholate in this model (−0.33 log_10_ CFU/mL/day). A total drug exposure of the area under the concentration-time curve from 0 to 24 h (AUC_0–24_) of 25 to 50 mg · h/L of APX2039 resulted in near-maximal antifungal activity. These data support the further preclinical and clinical evaluation of APX2039 as a new oral fungicidal monotherapy for the treatment of CM.

## INTRODUCTION

Cryptococcal Meningitis (CM) is a significant source of global morbidity and mortality. Current estimates are 220,000 cases and 181,000 deaths per year in individuals living with HIV/AIDS (1). The majority of infections are in Africa, but a significant number of cases continue to occur outside Africa due to progressively enlarging populations of immunocompromised hosts. Even in resource-rich health care systems, up to 15% of CM cases result in death (2), and this rate is much higher in resource-poor health care systems (1). The current treatment guidelines from the WHO include 7 days of intravenous amphotericin B deoxycholate (AMB) combined with oral flucytosine, followed by consolidation and then maintenance therapy with fluconazole (FLU) (3). Recently, with the report of the AMBITION study, the WHO also recommended induction therapy using a single dose of AmBisome (liposomal amphotericin B) at 10 mg/kg of body weight followed by 14 days of flucytosine at 100 mg/kg/day and fluconazole at 1,200 mg/day (4). In contrast, in resource-rich countries, a combination of a polyene plus flucytosine for the treatment of CM for 2 weeks remains the standard of care (5). Both amphotericin B and fluconazole target portions of the ergosterol pathway of Cryptococcus, while flucytosine inhibits RNA/DNA synthesis. Although effective, this combination poses problems of accessibility, compliance, toxicity, and clinical failures. Thus, there is an urgent need for new antifungal therapies that can effectively treat CM and especially oral drugs that can be more readily administered for induction, consolidation, and maintenance therapy.

Manogepix (APX001A) is the first compound in the new gepix class that demonstrates broad-spectrum *in vitro* antifungal activity (6). The *N*-phosphonooxymethylene prodrug fosmanogepix (APX001) demonstrated significant activity in a wide array of fungal infection models and is currently in clinical development for the treatment of *Candida*, Aspergillus, and rare mold infections (ClinicalTrials.gov identifiers NCT04148287, NCT03604705, and NCT04240886) (6). The cellular target of manogepix is the Gwt1 protein, which catalyzes an early step in glycosylphosphatidylinositol (GPI) anchor biosynthesis (7), from which the new class name “gepix” is derived. This process is important for anchoring proteins to the cell walls of fungi. Manogepix, the active moiety of the prodrug fosmanogepix, does not inhibit PIGW, the closest human homolog, even at concentrations >100-fold higher than the 50% inhibitory concentration (IC_50_) for inhibition of the Candida albicans and Aspergillus fumigatus Gwt1 proteins (7).

We previously reported on the efficacy of fosmanogepix and the analogous *N*-phosphonooxymethylene prodrugs of two other Gwt1 inhibitors, APX2020 and APX2039 (prodrugs APX2097 and APX2096, respectively), in murine models of cryptococcosis (8). In those studies, APX2096 was dosed at 60 mg/kg intraperitoneally (i.p.) 2 h after the administration of 100 mg/kg of 1-aminobenzotriazole (ABT), a pan-cytochrome P450 inhibitor that has been shown to increase systemic APX2039 exposure. In experiments where treatment with APX2096 was initiated 1 h after infection, the Cryptococcus neoformans H99 fungal burden was reduced by approximately 7 log_10_ CFU/g and 5 log_10_ CFU/g in the brain and lung tissue, respectively, after 7 days of therapy. Similarly, in a delayed (24 h postinfection) treatment model, the brain and lung fungal burdens were reduced by averages of 6.8 log_10_ CFU/g and 4.6 log_10_ CFU/g, respectively. The prodrug APX2097 (active moiety APX2020) was not effective in these models, which is consistent with the 8-fold-higher MIC of the active moiety APX2020 than APX2039 against C. neoformans var. *grubii* H99 (8). Although the mouse model is robust, the rabbit CM model offers an environment that more closely mimics that of the typical CM patient. The rabbits are immunosuppressed with hydrocortisone, which removes the anticryptococcal lymphocyte response, and the infection is established directly in the central nervous system (CNS) (9–11), whereas the mouse model utilizes immunocompetent mice and relies on systemic intravenous (tail vein) infection. Here, we present compelling evidence for the superior efficacy of orally administered APX2039, first in the mouse model and then in the rabbit model of CM, compared to FLU, AMB, and fosmanogepix.

## RESULTS

### APX2039 reduces the fungal burden in mice.

**FIG 1:**
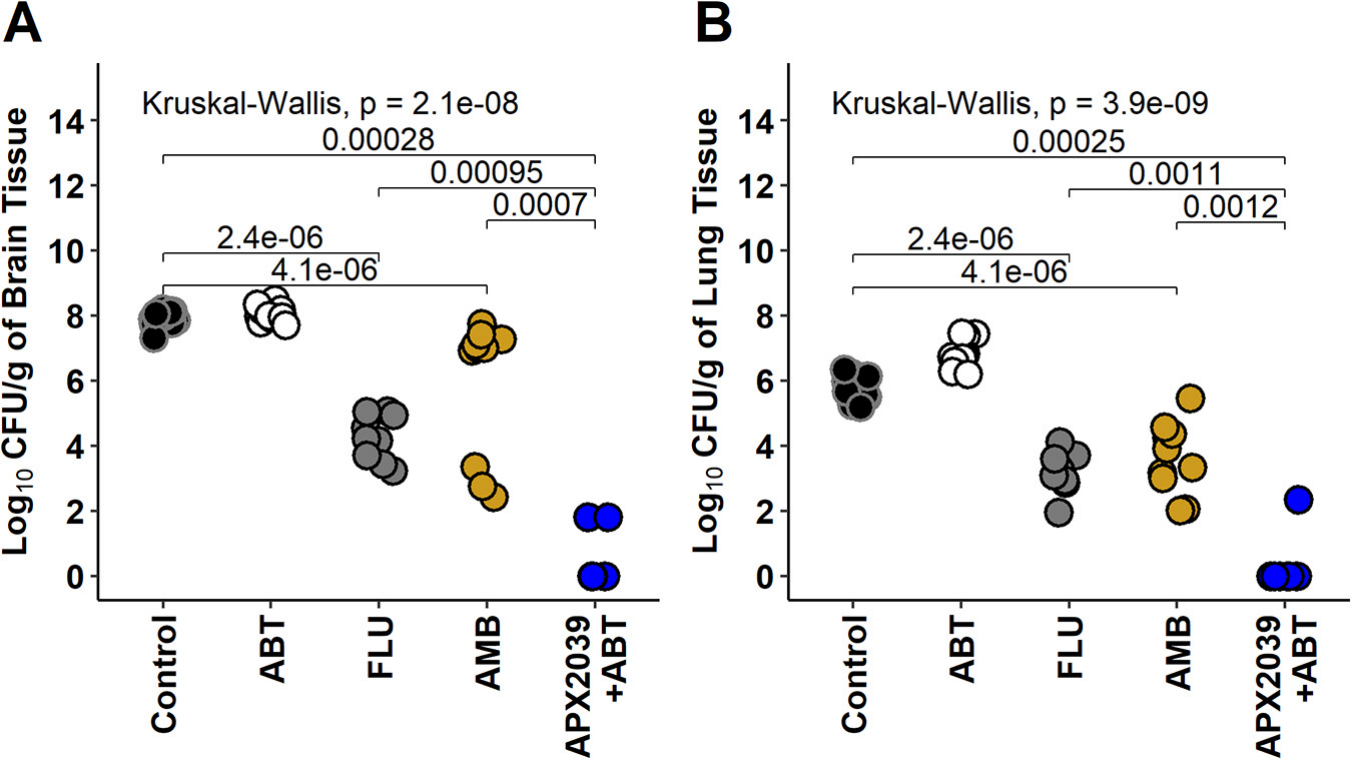
Mouse delayed-treatment model (log_10_ CFU per gram of mouse brain and lung tissue). Each point represents data from a single mouse. Data were analyzed with a Kruskal-Wallis test followed by a Wilcoxon rank sum test with Holm’s correction. Dosing groups were as follows: control (vehicle [p.o.]) (*n* = 14), ABT (50 mg/kg ABT [p.o., QD]) (*n* = 11), FLU (150 mg/kg fluconazole [p.o., QD]) (*n* = 9), AMB (3 mg/kg amphotericin B deoxycholate [i.p., QD]) (*n* = 10), and 60 mg/kg APX2039 (p.o., QD) plus 50 mg/kg ABT administered 2 h prior to dosing with APX2039 (*n* = 7). (A) Data from brain tissue. (B) Data from lung tissue.

The efficacies of 60 mg/kg of APX2039 plus 50 mg/kg of ABT (orally [p.o.], once a day [QD]) administered 2 h prior to dosing with APX2039, 3 mg/kg of AMB (i.p., QD), and 150 mg/kg of fluconazole (p.o., QD) were evaluated in a 24-h delayed-treatment model of disseminated cryptococcal disease. Mice were infected via tail vein injection with approximately 5 × 10^4^ cells of C. neoformans H99, and treatments were initiated at 24 h postinfection. Animals were euthanized on day 9 after 7 days of treatment. Vehicle controls with and without ABT were evaluated, and no significant differences were found between the two groups (Fig. 1). The fungal burden (log_10_ CFU per gram of tissue) in the lung and brain was significantly lower in all treatment groups. The reductions in the fungal burdens (mean log_10_ CFU/g control − mean log_10_ CFU/g treated) in the lung versus the vehicle control were 2.38, 1.36, and 4.45 log_10_ CFU/g for FLU, AMB, and APX2039, respectively. The reductions in the brain burden versus the vehicle control were 3.32, 0.82, and 6.53 log_10_ CFU/g for FLU, AMB, and APX2039, respectively. Treatment with APX2039 was significantly better than that with FLU or AMB in terms of reductions in fungal burdens in both the brain (*P *< 0.001) and lung (*P *≤ 0.002). Of note, a fungal burden below the limit of detection was observed only for APX2039. These data are consistent with a combination of a very potent direct antifungal effect of the drug as measured by a very low MIC and a favorable CNS pharmacokinetic/pharmacodynamic (PK/PD) profile (C. neoformans H99 MIC values previously observed for APX2039 [0.008 μg/mL] versus manogepix [0.25 μg/mL], AMB [0.25 μg/mL], and FLU [2 μg/mL] [8]).

### APX2039 is effective in a rabbit model of cryptococcal meningitis.

To evaluate efficacy in the rabbit CM model, male New Zealand White rabbits were inoculated with C. neoformans H99 (~1.4 × 10^6^ CFU) directly into the cisterna magna under sedation. Rabbits were immunosuppressed with daily hydrocortisone acetate. Treatments were initiated on day 2 postinfection and continued through day 14. Cerebrospinal fluid (CSF) was removed via a cisternal tap on days 2, 7, 10, and 14 postinfection to quantify the fungal burden. Animals were euthanized either on day 14 or when clinical symptoms required euthanasia, and the fungal burden in the brain tissue was assessed.

**FIG 2:**
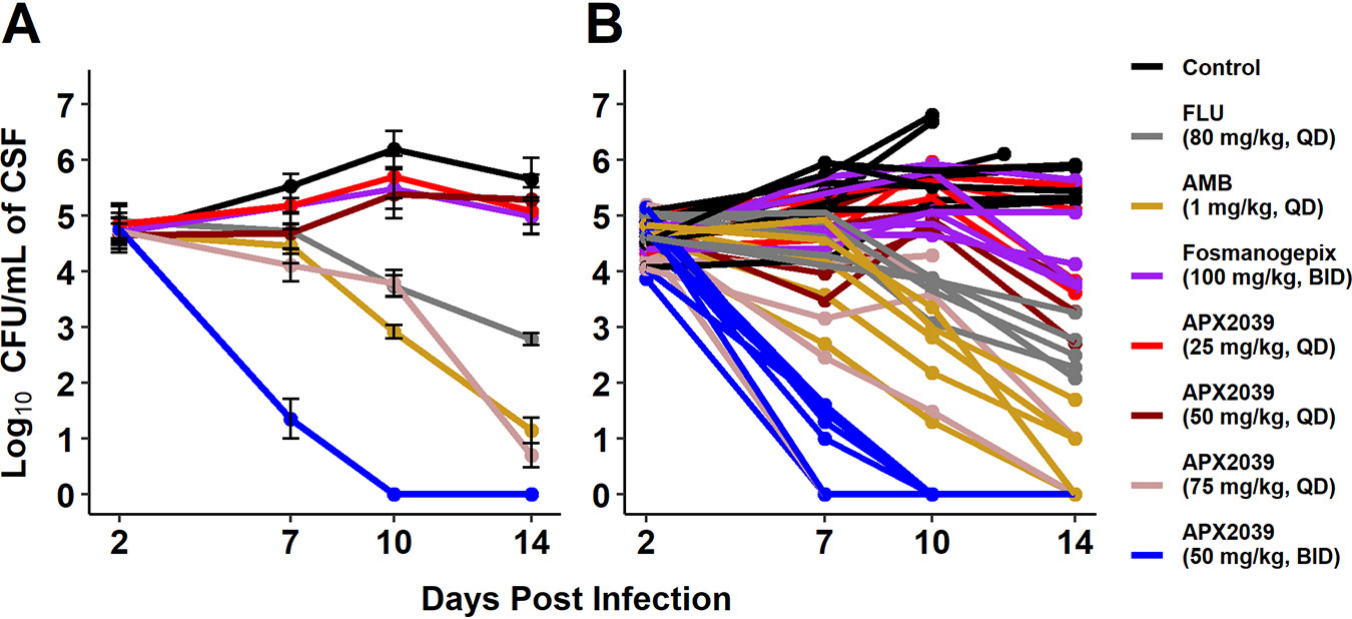
Average log_10_ CFU per milliliter of rabbit CSF over time. (A) CFU per milliliter of CSF data were averaged and then log_10_ transformed. Error bars are SEM. Data are pooled from three independent experiments (control, *n* = 9 for day 2, *n* = 11 for day 7, *n* = 9 for day 10, and *n* = 5 for day 14; fluconazole, *n* = 5 for all time points; amphotericin B, *n* = 5 for all time points; fosmanogepix, *n* = 6 for all time points; APX2039 [25 mg/kg, QD], *n* = 5 for days 2, 7, and 10 and *n* = 4 for day 14; APX2039 [50 mg/kg, QD], *n* = 4 for all time points; APX2039 [75 mg/kg, QD] *n* = 5 for day 2, day 7, and day 10 and *n* = 2 for day 14; APX2039 [50 mg/kg, BID], *n* = 8 for day 2, day 7, and day 10 and *n* = 7 for day 14). (B) All data from each rabbit plotted individually, with each line corresponding to a single rabbit.

The primary endpoint was the fungal burden in the CSF. To assess this, we collected CSF 2 days after the initial inoculation prior to treatment to determine the baseline yeast burden and then repeated the CSF collections on days 7, 10, and 14 postinfection (Fig. 2A and B). Reductions in CFU per milliliter for all treatment groups were statistically different from those for the control group, with the exception of APX2039 at 25 mg/kg QD (see Table S1 in the supplemental material). Rabbits administered APX2039 at 50 mg/kg BID demonstrated a rapid decrease in the fungal burden by day 7, and the fungal burdens were undetectable on days 10 and 14.

10.1128/mbio.02347-22.2 TABLE S1 Pairwise comparisons of the reductions in log_10_ CFU per milliliter of CSF. Download Table S1, DOCX file, 0.01 MB. Copyright © 2022 Giamberardino et al. 2022 Giamberardino et al. https://creativecommons.org/licenses/by/4.0/ This content is distributed under the terms of the Creative Commons Attribution 4.0 International license.

Daily treatment with APX2039 at 50 mg/kg BID resulted in a mean total change of −4.6 ± 0.44 log_10_ CFU/mL of CSF within 8 days of treatment. A dose response was observed for the APX2039 QD dosing groups, with a −2.7 ± 2.06 log_10_ reduction by day 14 in rabbits treated with 75 mg/kg, whereas 50 mg/kg resulted in a −0.72 ± 1.37 log_10_ CFU/mL reduction. Treatment with 25 mg/kg APX2039 was not effective and was equivalent to the vehicle control, as rabbits in this group had a small increase of 0.07 ± 1.30 log_10_ CFU/mL. Treatment with 100 mg/kg BID fosmanogepix was minimally effective and resulted in a −0.34  ± 0.86 log_10_ CFU/mL reduction. Treatment with AMB and FLU resulted in −3.9  ± 0.72 and −2.28 ± 0.57 log_10_ CFU/mL reductions, respectively. Rabbits that were either untreated or vehicle treated had an average increase of 1.17 ± 0.46 log_10_ CFU/mL versus day 2 values, starting at an average burden of 4.67 log_10_ CFU/mL and reaching 5.65 log_10_ CFU/mL by day 14.

Previous publications have reported the fungal clearance rate in log_10_ CFU per milliliter per day using a variety of approaches, including manually calculated slopes, linear models, and linear mixed-effects models (12–25). To compare the rates of reduction that we observed in these experiments with those in the existing literature, we employed three approaches. For the manual linear calculation, we compared the log-subtracted differences of the baseline and the final collection across the groups (Table 1) [fungal clearance = (log_10_ CFU/mL final − log_10_ CFU/mL baseline)/interval in days].

**TABLE 1:** Change in CFU in log_10_ CFU per milliliter of CSF

Treatment	Change in CFU/day				Total change in CFU during the study			
	Mean slope	No. of Rabbits	SD	SEM	Mean	SD	No. of Rabbits	SEM
Control	0.13	9	0.06	0.02	1.17	0.46	9	0.15
FLU (80 mg/kg, QD)	−0.19	5	0.05	0.02	−2.28	0.57	5	0.25
AMB (1 mg/kg, QD)	−0.33	5	0.06	0.03	−3.92	0.72	5	0.32
Fosmanogepix (100 mg/kg, BID)	−0.03	6	0.07	0.03	−0.34	0.86	6	0.35
APX2039 (25 mg/kg, QD)	0.02	5	0.13	0.06	0.07	1.30	5	0.58
APX2039 (50 mg/kg, QD)	−0.06	4	0.11	0.06	−0.72	1.37	4	0.69
APX2039 (75 mg/kg, QD)	−0.34	5	0.35	0.15	−2.70	2.06	5	0.92
APX2039 (50 mg/kg, BID)	−0.66	8	0.15	0.05	−4.60	0.44	8	0.16

**FIG 3:**
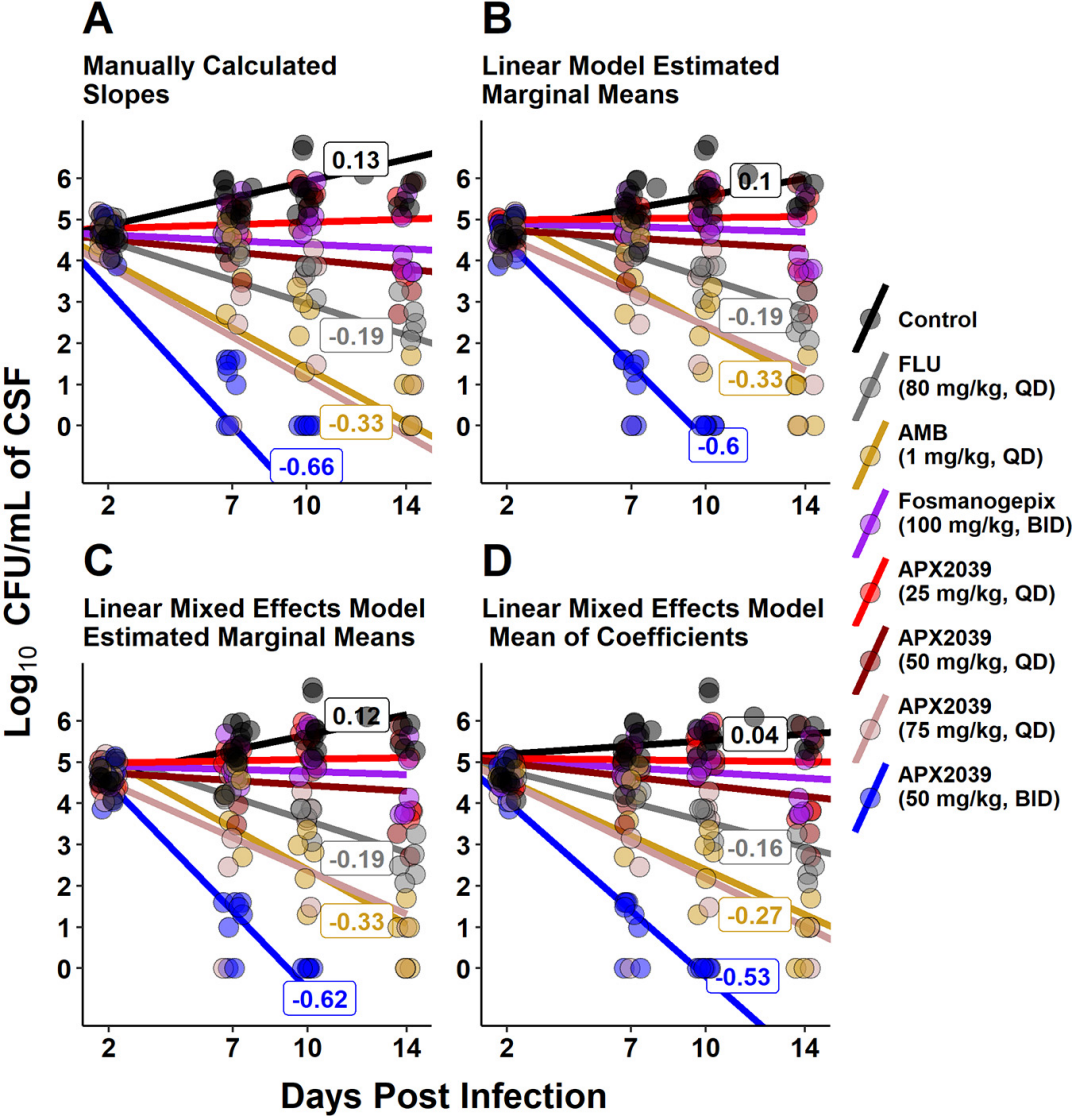
Log_10_ CFU per milliliter of rabbit CSF over time: plot of slopes and intercepts from a linear calculation, a linear model, and a linear mixed-effects model. Fungal clearance was calculated with four separate methods. Dots are log_10_ CFU per milliliter of CSF data collected during the study and are the same for each panel. Line labels are slopes of selected lines: control, FLU, AMB, and APX2039 (50 mg/kg, BID). (A) Slopes were manually calculated for each rabbit. The mean values of the slopes and intercepts were used to create the lines using the formula log_10_ CFU/mL = mean change in log_10_ CFU/mL/day · day postinfection + intercept mean. (B) Estimated marginal means from a linear model that includes an interaction between day postinfection and treatment (log_10_ CFU/mL ~ day postinfection · treatment). (C) Estimated marginal means from a linear mixed-effects model with day postinfection and treatment group as the fixed effects and rabbit with a random slope as the random effects [log_10_ CFU/mL ~ day postinfection · treatment + (0 + day postinfection|rabbit ID)]. (D) Means of coefficients extracted from a linear mixed model with only day postinfection as the fixed effect and random slopes for each rabbit [log_10_ CFU/mL ~ day postinfection + (0 + day postinfection|rabbit ID)].

Using this approach, APX2039 at 50 mg/kg BID demonstrated a remarkable reduction of −0.66 (standard deviation [SD], ±0.15) log_10_ CFU/mL/day, compared to −0.33 (SD, ±0.06) log_10_ CFU/mL/day for rabbits treated with AMB. We compared the means using a Kruskal-Wallis test followed by pairwise comparisons using a Wilcoxon rank sum test with Holm’s adjustment, with a *P* value of 0.054 for comparisons of APX2039 at 50 mg/kg BID to both AMB and FLU (Fig. 3A).

Using a linear model, we examined the effect of treatment and day postinfection on the log_10_ CFU per milliliter of CSF. We tested two models, one with an interaction of treatment group and day and one without the interaction, where treatment was a cofactor and day was a covariate. The model was created using all experiment data, and the coefficients within each model were then compared to the control values. We performed pairwise comparisons of the estimated marginal means for linear model 1 (log_10_ CFU/mL ~ day + treatment) and linear model 2 (log_10_ CFU/mL ~ day · treatment [includes interaction]).

In the linear model without the interaction, the overall slope was −0.13 (95% confidence interval [CI], −0.17 to 0.09) log_10_ CFU/mL/day. Treatment with AMB reduced the log_10_ CFU per milliliter by −1.33 (95% CI, −2.88 to 1.66) units. Treatment with APX2039 at 50 mg/kg BID resulted in a far-larger effect, with a −3.47 (95% CI, −4.07 to −2.88)-log_10_ CFU/mL reduction (FLU, *P* ≤ 0.0001; AMB, *P* = 0.014 [by pairwise comparisons]). We then incorporated an interaction to more directly probe the slope of the groups and the effect of group for each unit change in days within the model (Fig. 3B). We calculated the estimated marginal means and then extracted the slopes. APX2039 at 50 mg/kg BID reduced the log_10_ CFU/mL by −0.60 (95% CI, −0.69 to −0.50) units per day, compared to reductions of −0.19 (95% CI, −0.27 to −0.12) and −0.33 (95% CI, −0.40 to −0.26) log_10_ CFU/mL/day for FLU and AMB, respectively (*P < *0.0001 by pairwise comparisons of estimated marginal means for both FLU and AMB).

Next, to align our findings with the methodology employed previously by Molloy et al. (14), we used a linear mixed-effects model to model the fungal burden in CSF across treatment groups, with an interaction between day postinfection and treatment group as the fixed effects and individual variation in each rabbit as the random effect, along with random slopes for each rabbit [log_10_ CFU/mL ~ day postinfection · treatment + (0 + day|rabbit ID)]. Because we infected all rabbits at the same time with the same inoculum, we included a fixed intercept. Analyses of the models with a random intercept did not improve the model (Fig. 3C).

We calculated the estimated marginal means and then extracted the slopes. FLU and AMB accounted for daily decreases of −0.19 (95% CI, −0.27 to −0.11) and −0.33 (−0.41 to −0.25) log_10_ CFU/mL for each day postinfection, respectively (Table 2). Oral APX2039 at 50 mg/kg BID showed a more rapid reduction than AMB or FLU, at −0.62 (95% CI, −0.71 to −0.54) log_10_ CFU/mL/day (Table 2), which compares very favorably with a good clinical outcome in humans associated with a reduction of approximately −0.4 log_10_ CFU/mL/day in the CSF (14). Pairwise comparisons of the estimated marginal means between APX2039 at 50 mg/kg BID and FLU and AMB had *P* values of <0.0001 (Table 3).

**TABLE 2:** Slopes from a linear mixed-effects model for CSF in the rabbit model of cryptococcal meningitis

Treatment	Change in Log_10_ CFU/mL/day			
	Slope	SE	Lower confidence level	Upper confidence level
Control	0.12	0.03	0.06	0.19
Fluconazole	−0.19	0.04	−0.27	−0.11
Amphotericin B	−0.33	0.04	−0.41	−0.25
Fosmanogepix	−0.02	0.04	−0.09	0.06
APX2039 (25 mg/kg, QD)	0.01	0.04	−0.07	0.09
APX2039 (50 mg/kg, QD)	−0.04	0.05	−0.13	0.05
APX2039 (75 mg/kg, QD)	−0.27	0.05	−0.36	−0.17
APX2039 (50 mg/kg, BID)	−0.62	0.04	−0.71	−0.54

**TABLE 3:** Selected pairwise contrasts of estimated marginal means

Contrast	*P* value
Control-APX2039 (50 mg/kg, BID)	<0.0001
Fluconazole-APX2039 (50 mg/kg, BID)	<0.0001
Amphotericin B-APX2039 (50 mg/kg, BID)	<0.0001
Control-APX2039 (75 mg/kg, QD)	<0.0001

One final statistical approach to examine the change in the CSF fungal burden used a linear mixed-effects model but did not include treatment as a fixed effect [log_10_ CFU/mL ~ day postinfection + (0 + day postinfection|rabbit ID)] (Fig. 3D). This created a single slope for all of the data, and the only adjustments to the slope or intercept were from the individual rabbits.

The coefficients for each rabbit were then extracted and compared using one-way analysis of variance (ANOVA) (*P *< 0.001), and pairwise comparisons of means were then performed using Tukey’s honestly significant difference test. Using this approach, APX2039 at 50 mg/kg BID had an average reduction of −0.53 (SEM [standard error of the mean] = 0.24) log_10_ CFU/mL/day, compared to −0.15 (SEM = 0.1) log_10_ CFU/mL/day for FLU and −0.27 (SEM = 0.03) log_10_ CFU/mL/day for AMB. Regardless of the approach used to evaluate the rate of fungal clearance in the CSF, APX2039 at 50 mg/kg BID resulted in −0.2 log_10_ CFU/mL/day greater clearance than either AMB or FLU (Table 4).

**TABLE 4:** Selected pairwise comparisons of coefficients from a linear mixed-effects model

Comparison	*P* value
Control-APX2039 (50 mg/kg, BID)	<0.0001
Fluconazole-APX2039 (50 mg/kg, BID)	<0.0001
Amphotericin B-APX2039 (50 mg/kg, BID)	0.0001
Control-APX2039 (75 mg/kg, QD)	<0.0001

**FIG 4:**
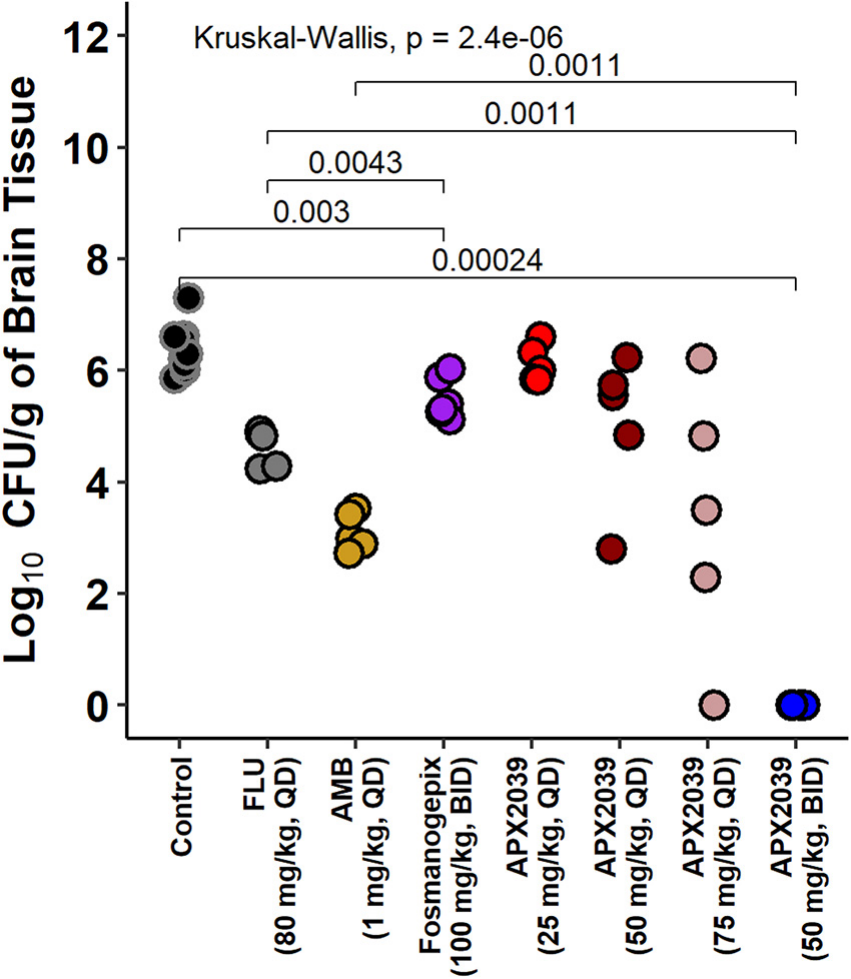
Brain tissue fungal burden in the rabbit model of cryptococcal meningitis. Each point represents data from a single rabbit. Group effects were tested using a Kruskal-Wallis test. Pairwise comparisons were made using a Wilcoxon rank sum test with Holm’s adjustment (control, *n* = 10; FLU, *n* = 5; AMB, *n* = 5; fosmanogepix, *n* = 6; APX2039 at 25 mg/kg QD, *n* = 5; APX2039 at 50 mg/kg QD, *n* = 5; APX2039 at 75 mg/kg QD, *n* = 5; APX2039 at 50 mg/kg BID, *n* = 8).

To examine the burden of yeasts in the rabbit brain, we collected brain tissue after euthanasia. Brains harvested on day 14 demonstrated mean reductions versus the control of −1.8 and −3.4 log_10_ CFU/g tissue for FLU and AMB, respectively, while a mean reduction of −6.6 log_10_ CFU/g was observed for APX2039 (Fig. 4). After the administration of APX2039 at 50 mg/kg BID, the burdens of yeasts in both the cerebellum/brainstem and cerebrum were below the limit of detection (data not shown). When comparing the total yeast burdens in the brain, treatment with APX2039 showed superiority over both FLU and AMB (adjusted *P* = 0.027 for both comparisons by a Wilcoxon rank sum test). Rabbits treated with APX2039 at 50 mg/kg BID had undetectable CFU in the vitreous humor, but the statistical comparisons to FLU and AMB did not have an adjusted *P* value of <0.05 (Fig. S1). Fosmanogepix was not effective in reducing the fungal burden in brain tissue at day 14 relative to the control (adjusted *P* = 0.06) and performed worse than either FLU or AMB (adjusted *P* = 0.082 for both by a Wilcoxon rank sum test). Fosmanogepix was able to reduce the fungal burden in the vitreous humor relative to the control but did not perform as well as FLU (Fig. S1).

10.1128/mbio.02347-22.1 FIG S1 Fungal burden in the vitreous humor. Download FIG S1, TIF file, 1.4 MB. Copyright © 2022 Giamberardino et al. 2022 Giamberardino et al. https://creativecommons.org/licenses/by/4.0/ This content is distributed under the terms of the Creative Commons Attribution 4.0 International license.

### PK analysis and drug concentrations.

**FIG 5:**
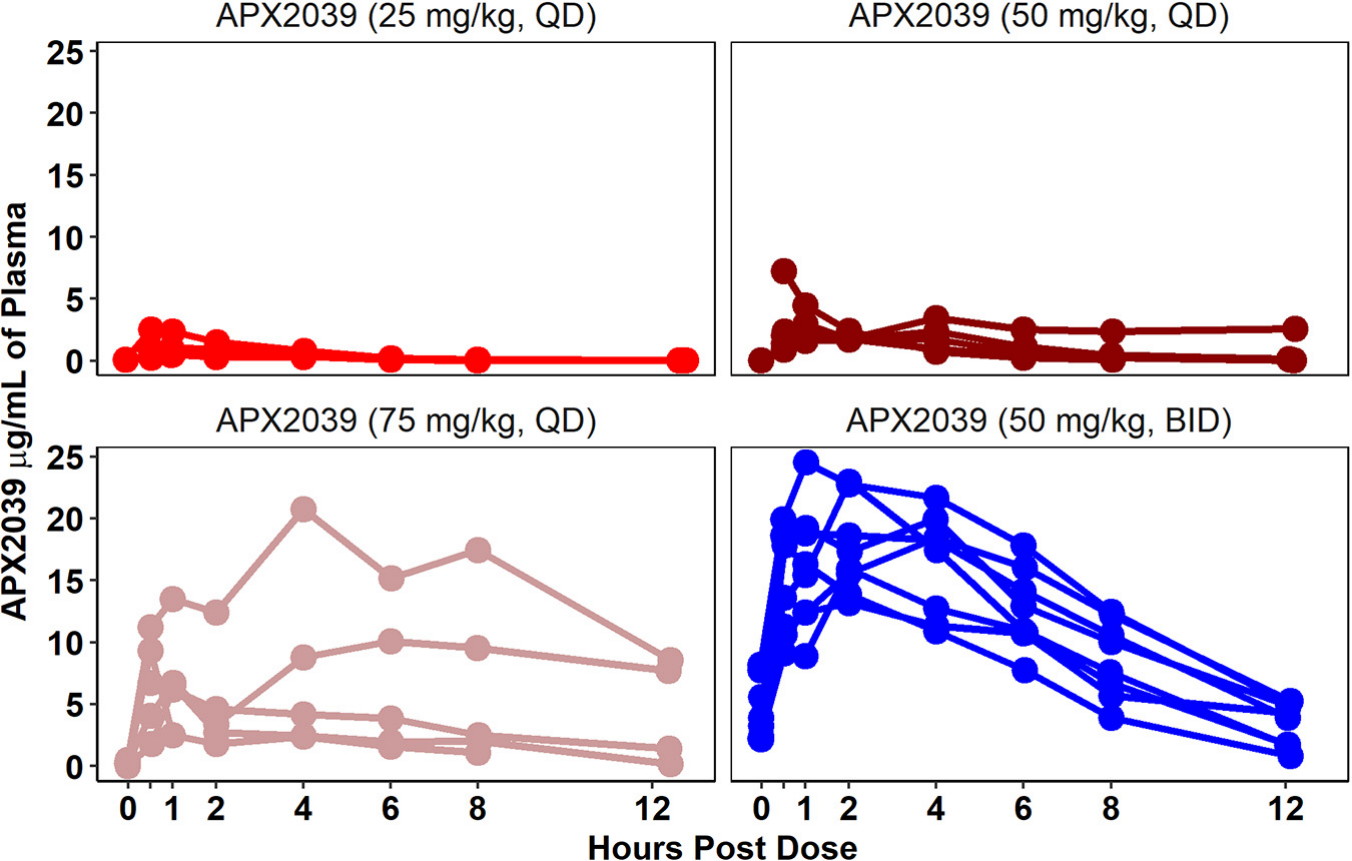
Plasma levels of APX2039. Each line shows data from a single rabbit. Plasma was collected from each rabbit for the following treatments: APX2039 at 25 mg/kg QD (*n* = 5) (top left), APX2039 at 50 mg/kg QD (*n* = 5) (top right), APX2039 at 75 mg/kg QD (*n* = 5) (bottom left), and APX2039 at 50 mg/kg BID (*n* = 8) (bottom right).

**FIG 6:**
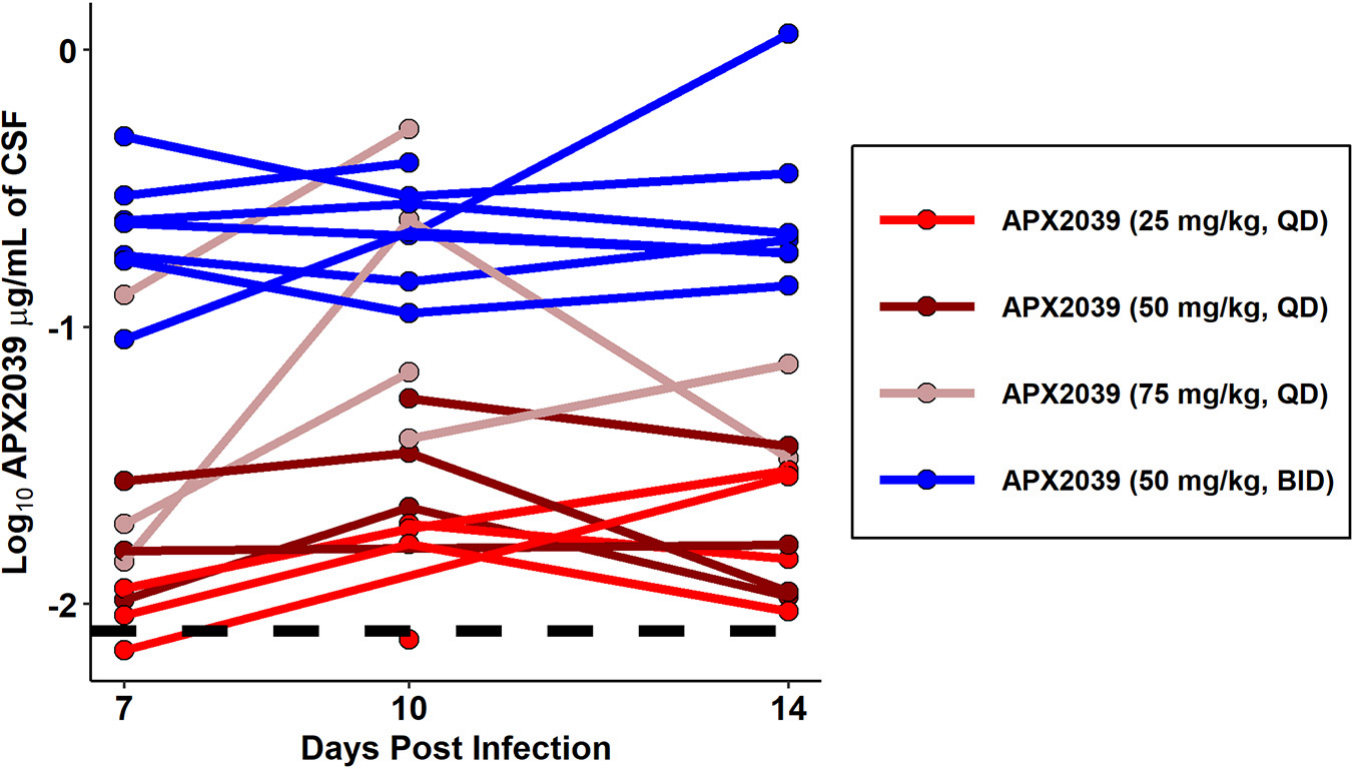
Levels of APX2039 in rabbit CSF. Concentrations of APX2039 in micrograms per milliliter of CSF were quantified by LC-MS. Each line shows data from a separate rabbit. All CSF samples were collected 1 to 2 h after dosing with APX2039. The black dotted line is the log_10_-transformed value of the MIC of APX2039 (0.008 μg/mL).

To investigate the PK of APX2039 in the rabbit model of CM, we performed serial blood collections after dosing on day 8 postinfection, after 6 days of dosing. The PK parameters are shown in Table 5. The results across the groups can be seen in Fig. 5, with 50-mg/kg BID dosing having higher levels of APX2039 than with 25, 50, or 75 mg/kg QD. Although the PK index that best links drug exposure with antifungal efficacy has not been formally investigated for APX2039, the efficacy of the related Gwt1 inhibitor manogepix was previously shown to be the area under the concentration-time curve (AUC)/MIC for C. albicans, and this metric was used in the PK/PD analyses. The PK was linear. Levels of APX2039 in the CSF were well above the MIC for rabbits dosed at 50 mg/kg BID and 75 mg/kg QD and closer to the MIC for rabbits dosed with either 25 mg/kg QD or 50 mg/kg QD. This finding aligns with the lower fungal burdens in CSF for both the 50-mg/kg BID and 75-mg/kg QD dosing groups (Fig. 6).

**Table 5:** Values for each parameter estimated from the rabbit population pharmacokinetic/pharmacodynamic-linked model

Parameter (Unit)	Mean	Median	SD
K_a_ (h^-1^)	14.452	13.778	12.135
SCL (L/h)	5.869	4.644	4.654
V (L)	15.002	13.204	10.766
K_cp_ (h^-1^)	7.432	7.434	5.713
K_pc_ (h^-1^)	14.103	11.101	9.573
K_gmax_ (Log_10_ CFU/mL/h)	0.114	0.095	0.086
H_g_	9.218	7.271	7.635
C_50g_ (mg/L)	5.841	5.748	3.387
Popmax (CFU/mL)	255386.168	202038.155	315859.857
K_kmax_ (Log_10_ CFU/mL/h)	0.321	0.235	0.244
H_k_	16.733	16.081	11.843
C_50k_ (mg/L)	4.638	3.150	3.745
IC (CFU/mL)	540.496	510.244	329.735

### PK/PD analysis.

The PK-PD model fitted the data well. The coefficients of determination for the linear regression of observed-predicted values for the plasma and fungal density in the CSF were 0.94 and 0.85%, respectively, after the Bayesian step. The estimates of central tendency and dispersion for the parameters from the PK/PD model are summarized in Table 5.

**FIG 7:**
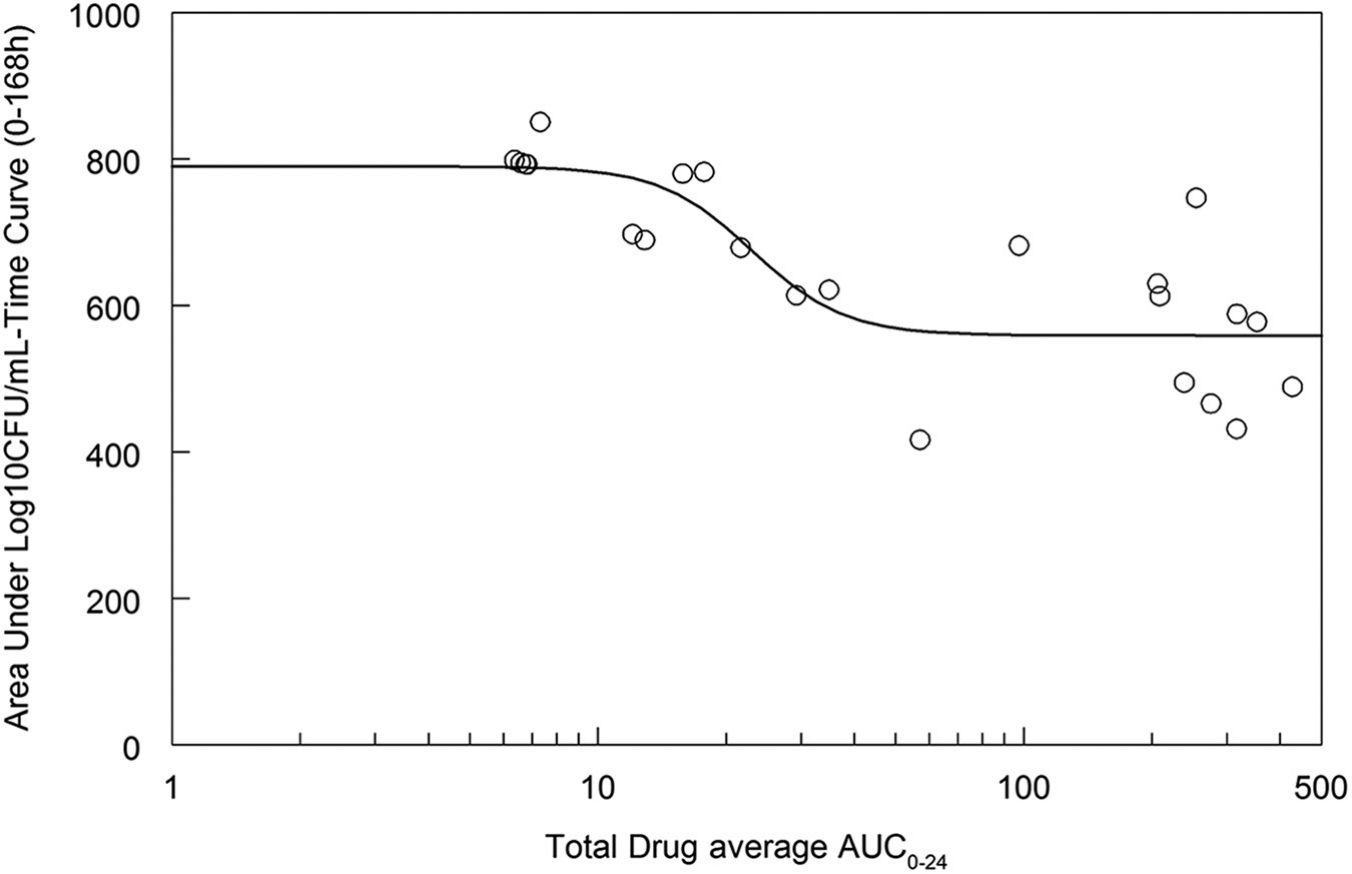
Average total drug AUC_0–24_ and decline in the log_10_ CFU per milliliter as estimated by the area under the fungal density (log_10_ CFU per milliliter)-versus-time curve for each rabbit.

The relationship between the average total drug AUC from 0 to 24 h (AUC_0–24_) and the decline in log_10_ CFU per milliliter as estimated by the area under the log_10_ CFU per milliliter-versus-time plots is shown in Fig. 7. Near-maximal antifungal activity was achieved with a total drug AUC_0–24_ of 25 to 50 mg · h/L and a total drug AUC/MIC ratio of 3,125 to 6,250.

## DISCUSSION

CM was uniformly fatal until treatment with amphotericin B deoxycholate was developed in the late 1950s (16). A second major improvement was identified with the successful use of amphotericin B deoxycholate and flucytosine in combination therapy, which reduced toxicity and shortened the treatment course (17). In the early 1990s, the approval of fluconazole allowed a safe, oral anticryptococcal treatment that was successful in managing some cases of CM, but it was clear that fluconazole monotherapy would not be consistently successful (18). Therefore, with three drugs but no uniformly successful monotherapy, a landmark article in 1997 delineated CM management into 3 stages: induction, consolidation, and suppression (19). Since that critical study, many resource-available health care systems have established specific guidelines using a combination of amphotericin B, or a lipid formulation of AMB, plus flucytosine followed by various doses of fluconazole. Over the last 3 decades, it has been clearly shown with the use of quantitative CSF yeast cultures that it is important to achieve rapid effective fungicidal activity (EFA) with treatment to efficiently reduce the fungal burden and, thus, improve patient outcomes (13, 14). Furthermore, it was shown that fluconazole is most effective after the yeast burden is already substantially reduced. Mechanistically, this likely occurs because fluconazole acts as a fungistatic drug in the central nervous system, which may be due in part to the plasticity of the cryptococcal genome under stress and yeast drug selection. Fluconazole heteroresistance and tolerance frequently occur in monotherapy with high-burden yeast infections, and thus, it is not consistently effective as monotherapy (18).

There have been only small incremental improvements in the therapy of CM over the last 30 years, for instance, refinements in drug formulations such as lipid-based amphotericin B formulations, other combination therapies (amphotericin B and fluconazole, flucytosine and fluconazole, and triple therapy), and even a single dose of AmBisome (liposomal amphotericin B) on an azole and flucytosine base. Unfortunately, the search for an effective new anticryptococcal drug class has not been rewarding. For example, research on repurposed drugs like sertraline and tamoxifen failed to show a benefit in CM (20–22). Simply, this life-threatening common fungal CNS infection needs better therapies.

Our laboratory has studied many antifungal compounds in the immunosuppressed rabbit model, which uses the impact of drugs on fungicidal activity in the subarachnoid space as an endpoint, similar to human studies. By allowing the model to establish the infection over 2 days, at a yeast burden similar to that observed in heavily infected humans prior to treatment, the rabbit model presents a very high barrier to drug success. A similar endpoint of EFA in the subarachnoid space was adopted in human clinical trials, and the EFA measured early in the treatment course in rabbit and human CSF was correlated with the outcome (23, 24). The rabbit has thus been a reasonable surrogate for outcomes that might occur in humans. For example, despite very low CSF levels of amphotericin B, monotherapy with this polyene can produce CSF fungicidal activity (11). In addition, a polyene can have a positive interaction with azole therapy in reducing CSF yeast counts (10). Furthermore, the rabbit model showed that amphotericin B lipid formulations could work in CM if there was careful attention paid to the dosing of the lipid formulation (25). With the ability to administer high-dose AMB lipid formulations, the hypothesis of whether liposomal amphotericin B could be effectively given in an intermittent dose schedule compared to daily dosing was tested, and it was found to possess similar efficacies with both schedules (26). These findings allowed investigators to test the efficacy of the intermittent dosing of liposomal amphotericin B in humans, and it culminated in the recent randomized controlled AMBITION trial with 844 patients that showed the noninferiority of one high dose of liposomal amphotericin B (10 mg/kg) on a base of azole and flucytosine compared to daily polyene therapy (4, 27). The rabbit model predicted this outcome (26). Also, a series of azoles were tested in the rabbit model, including fluconazole (28), posaconazole (29), and isavuconazole (30). Similar to monotherapy in humans, these azoles all tend to be fungistatic or possess a slow fungicidal response in the rabbit. Thus, with serial CSF quantitative yeast measurements, a CNS site of infection, severe cell-mediated host immunosuppression, and a track record with drugs also used in human disease that recapitulates or predicts outcomes in humans, this rabbit model can be a critical link between *in vitro* susceptibility testing and possible human disease outcomes for testing new potential anticryptococcal agents.

In the current rabbit study, we demonstrated that the oral administration of 50 mg/kg APX2039 BID resulted in a rapid reduction of the fungal burden in the CSF, and we further showed the concomitant clearance of the yeast in brain tissue. These results provide strong evidence for oral APX2039, a member of the gepix class, as a potential new therapy for CM. We also examined the efficacy of fosmanogepix, which is presently in clinical trials for the treatment of *Candida*, Aspergillus, and rare mold infections. However, APX2039 was specifically chosen from a series of Gwt1 inhibitors because of its improved *in vitro* anticryptococcal activity compared to the active moiety, manogepix (0.008 μg/mL versus 0.25 μg/mL) (7). It was hypothesized that the higher potency of APX2039 would make it an ideal drug for the treatment of cryptococcal meningoencephalitis in the quest for rapid EFA in the host CNS site if the PK/PD were supportive, and this study confirmed this assumption.

APX2039 at 50 mg/kg BID produced a remarkable rate of CSF clearance of between −0.53 and −0.66 log_10_ CFU/mL/day using several calculation methods. In this model, it significantly outperformed fluconazole, amphotericin B, and fosmanogepix, for which dosing regimens were chosen to give rise to exposures in rabbits that are similar to those achieved clinically for each of the drugs. Furthermore, the rate of clearance was higher than the rate of successful yeast reduction of log_10_ CFU per milliliter per day in humans, where the most potent regimen of amphotericin B and flucytosine was found to be approximately −0.4 to −0.5 log_10_ CFU/mL/day (13, 14). Impressively, by 8 days of treatment with oral APX2039 alone, both the CSF and brain tissue had undetectable levels of yeasts. This rate of fungal clearance has not been observed previously with azole or polyene treatments in our model, and even high-dose liposomal amphotericin B (5 mg/kg/day) for 3 days could not consistently sterilize the CSF in the model in a week (26).

Although the burden of yeasts was undetectable in the brain tissue at the time of necropsy after 50 mg/kg APX2039 BID, we did not determine how early this occurs or if the tissue was sterilized with no yeast survival. Future experiments may determine the kinetics of clearance of Cryptococcus from the brain and if the clearance effect persists after the cessation of the APX2039 therapy for several weeks without treatment. These findings might gauge how long induction therapy with APX2039 is needed for reliable tissue sterilization and help predict future studies in humans for the length of drug exposure prior to consolidation or suppression stages or even if these secondary stages are necessary if no viable yeasts survive after 10 to 14 days of treatment.

In summary, this is a new potential oral antifungal agent with extremely potent *in vitro* anticryptococcal activity against Cryptococcus neoformans that is clearly translated into rapid EFA in several mammalian models of cryptococcosis. Importantly, the impressive activity of APX2039, which inhibits the novel Gwt1 target, suggests that oral monotherapy may be a viable option, without the need for a second antifungal agent or prolonged therapies. There are now PK/PD relationships that can help predict initial and maintenance dosing goals in humans. These results have set the stage for studies of this drug in humans that could potentially revolutionize the treatment of CM.

## MATERIALS AND METHODS

### Animals.

All animal experiments were performed in accordance with Duke University IACUC guidelines and approval.

### Mice.

Male CD-1 mice, weighing 22 to 24 g, were obtained from Charles River Laboratories International. They were infected with approximately 5 × 10^4^ cells of C. neoformans (strain H99) via tail vein injection. Daily treatment was initiated 24 h after infection and continued for 7 days, with 50 mg/kg ABT (p.o.) being administered 2 h prior to each APX2039 (60 mg/kg, p.o.) dose, AMB (3 mg/kg, i.p.), fluconazole (150 mg/kg, p.o.), ABT alone (50 mg/kg, p.o.), or the vehicle (5% dextrose), as previously described (7). Mice were euthanized 24 h after the last dose, and the brain and left lung were used to quantify the fungal burdens.

### Rabbits.

Male New Zealand White rabbits, weighing between 2.3 and 3.1 kg, were purchased from Robinson Services Incorporated (Winston-Salem, NC). They were administered hydrocortisone acetate at 5 mg/kg intramuscularly (i.m.) daily, beginning 1 day prior to infection. If a rabbit showed disabling neurological signs, it was euthanized prior to the study endpoint.

The inoculum was prepared as previously described (31). The concentration was adjusted to approximately 3.3 × 10^6^ cells/mL in phosphate-buffered saline (PBS), and the rabbits were inoculated with 0.3 mL of the suspension. Rabbits were sedated with ketamine (30 mg/kg) and xylazine (3 mg/kg) i.m. Next, 0.3 mL of the inoculum was injected into the cisterna magna. The rabbit was then administered yohimbine at 0.2 mg/kg intravenously (i.v.) (ZooPharm) to antagonize the anesthesia. Rabbits were recovered with fluids and supplemental heat.

### APX2039, fosmanogepix, AMB, and FLU preparation and dosing.

APX2039 was reconstituted to a 12.5-mg/mL solution suspended in 10% *N*,*N*-dimethylacetamide (DMA), 20% propylene glycol (PG), and 40% polyethylene glycol 400 (PEG 400) (pH 2 to 3). When precipitate was visible, aliquots were sonicated for 20 min in a 37°C water bath immediately prior to dosing. Fosmanogepix was supplied as a dry powder and stored at −20°C. The powder was reconstituted to a 25-mg/mL solution in 5% dextrose, with a pH of between 7.5 and 8.0, and aliquoted. Fluconazole (Rising) was purchased as a dry powder for oral administration at a concentration of 40 mg/mL. The powder was reconstituted according to the manufacturer’s instructions. Fresh aliquots were prepared each day.

The fosmanogepix, APX2039, and fluconazole solutions were administered by oral gavage. Administration started on day 2 postinfection and continued through the day of euthanasia. For doses that occurred on CSF collection days, with the exception of day 2 postinfection, the drug was administered prior to CSF collection.

Amphotericin B deoxycholate (XGen Pharmaceuticals) was purchased as a lyophilized powder. The powder was reconstituted using sterile USP-grade water and adjusted to a final concentration of 2.5 mg/mL in 5% dextrose using a 50% dextrose stock. The rabbit’s ear was numbed with lidocaine-prilocaine cream before dosing. The drug was administered through the marginal ear veins.

### CSF collection and CFU quantification.

CSF was collected on days 2, 7, 10, and 14 postinfection. If a rabbit needed to be euthanized before the study endpoint period of 14 days, then a final CSF sample was collected. For CSF collection, the sedation methods were identical to the methods used for the inoculations. The rabbits were given yohimbine and recovered according to the same protocol as that used for the inoculation.

Samples were plated onto yeast extract-peptone-dextrose (YPD) agar containing chloramphenicol (100 μg/mL) in a petri dish. For dilutions, 0.1 mL of the CSF was serially diluted (1:10) in PBS. The petri dishes were incubated for 3 to 5 days at 30°C. The CFU were counted, and the CFU per milliliter were calculated by multiplying the colonies counted by the dilution factor/0.1 mL. The remaining CSF that was not used for CFU quantification was centrifuged at 2,000 relative centrifugal force (rcf). The supernatant was collected and stored at −80°C for CSF drug level measurements by liquid chromatography-mass spectrometry (LC-MS) analysis.

### PK/PD blood collection and plasma processing.

On day 8 postinfection, PK/PD blood collection was performed. The ears were numbed with lidocaine-prilocaine cream. The rabbits were lightly sedated with acepromazine at 1 to 2 mg/kg subcutaneously. A 1.5-in. 21- to 23-gauge catheter was then inserted into the ear artery. Blood samples were collected in K_2_EDTA tubes (BD). The samples were centrifuged at 2,000 rcf for 15 min at 4°C and stored in 2-mL screw-cap tubes at −80°C for LC-MS analysis.

### Euthanasia and tissue collection.

Rabbits were sedated with ketamine at 40 mg/kg i.m. and xylazine at 5 mg/kg i.m. CSF was collected as described above. One milliliter of pentobarbital-phenytoin (Euthasol; Virbac, Westlake, TX, USA) was administered intravenously. A final blood sample was collected from the ear artery prior to the administration of pentobarbital-phenytoin or via cardiac puncture immediately after pentobarbital-phenytoin administration. The aqueous humor, vitreous humor, meninges, and brain were collected and either frozen on dry ice for later LC-MS analysis or homogenized in PBS for the quantification of the fungal burden.

The tissues from the right side were placed into 8-mL screw-cap tubes (Sarstedt) and homogenized using a Pro Scientific P200 homogenizer, or they were placed into tubes that were prefilled with homogenization beads (Precellys; Bertin Corp., France) and homogenized using a Precellys instrument at 6,500 rpm for 30 s.

### LC-MS analysis.

For all tissues, one of two internal standard solutions (ISSs) was used for homogenization or dilution: either 50% acetonitrile in water containing 10 nM warfarin and 1 μM natamycin or 50% acetonitrile in water containing 10 nM warfarin, 1 μM natamycin, and 20 μM *d*_6_-APX2039 internal standard.

Rabbit brain tissue samples were homogenized using Precellys lysing kits (Bertin, France) in the ISS at 10,000 rpm for 10 s for 3 cycles, with 60-s pauses between cycles, at 4°C. The homogenates were centrifuged at 13,000 rcf for 5 min at 4°C. The cleared lysates were stored at −80°C before analysis. The tissues were run in two batches, with curve ranges of 20 nM to 10 μM and 2.2 nM to 2.22 μM. Samples were diluted up to 1000-fold in 50% acetonitrile before the LC-tandem MS (MS/MS) analysis. The calibration curve was made using APX2039 dissolved in dimethyl sulfoxide (DMSO) and then diluted in either a naive rabbit brain homogenate or the ISS.

The CSF samples were mixed 10:1 with the ISS and then run with curve ranges of 1.06 nM to 20 μM or 1 nM to 10 μM. Samples were diluted up to 10-fold in the ISS. The plasma samples were diluted up to 400-fold in the ISS. The curves ranged from 20 nM to 10 μM or 30 nM to 20 μM. The calibration curves were made with the diluted ISS in PBS or rat plasma.

The analytical LC-MS/MS method, using selected reaction monitoring (SRM), was performed on an Acquity ultraperformance liquid chromatography (UPLC) system coupled to a Xevo TQ-S mass spectrometer. Mobile phase A consists of 5 mM ammonium acetate containing 0.2% formic acid (FA) and 0.025% heptafluorobutyric acid (HFBA) in water, while mobile phase B is acetonitrile with 0.2% FA. A 2.1-mm by 50-mm BEH Shield RP18 column (Waters) with a flow rate of 0.50 mL/min and a column temperature of 35°C was utilized. The electrospray source conditions were 2.5 kV, a desolvation temperature of 400°C, desolvation gas flow at 1,000 L/h, cone gas at 150 L/h, and 7.0 × 10^5^ Pa of nebulizer gas. MS/MS transitions for the most intense product ions were selected from an infusion experiment, and collision energy optimization was performed using LC-MS/MS and Skyline v20.0 (www.skyline.ms). Data analysis was performed with Skyline-daily 20.2 using the small-molecule mode, including peak extraction and quantification, linear regression, and concentration calculations. APX2039 was quantified versus a linear regression against a calibration curve with 1/*x*^2^ weighting, excluding calibration points that were outside 15% bias except at the lower limit of quantification (LLOQ) (20%).

### Brain and eye tissue CFU quantification.

The homogenates from the right eye and the right brain hemisphere were used for quantification. Samples were serially diluted in PBS, plated onto petri dishes containing YPD plus chloramphenicol (100 μg/mL), and incubated for 3 to 5 days at 30°C. The CFU per milliliter were obtained by multiplying the CFU counted by the dilution factor and then dividing this value by 0.1 mL. The total volume was obtained by adding the net weight of the tissue (the density was assumed to be 1 g/mL) to the volume of PBS that was in the tube. These two values, CFU per milliliter and total milliliters, were multiplied to obtain the total CFU. This value was then divided by the net weight in grams to yield the CFU per gram.

### Data analysis.

Data were cleaned using tidyverse (version 1.3.1) (32) and stringr (version 1.4.0) (33). Statistical analyses were performed in R (34) version 4.1.0. For fungal clearance calculations, if a rabbit had CFU per milliliter equal to zero prior to day 14, and the counts remained at zero, then only the data through the first instance of the CFU per milliliter being equal to zero were used for calculations.

To calculate the linear slopes for the change in the CFU per milliliter of CSF, the numerator was calculated by subtracting the day 2 postinfection log_10_ CFU per milliliter from the log_10_ CFU per milliliter for the first day that the CFU counts equaled zero, or if the rabbit had detectable CFU counts at all time points, then the day of the last sample was used. If the day 2 postinfection CFU sample was not usable, the rabbit was excluded from the calculations in the table. The denominator was calculated by subtracting 2 from the day of the final CFU count.

Pairwise comparisons of the linear mixed-effects model with the interaction between day postinfection and treatment group, log_10_ CFU/mL ~ day · treatment + (0 + day|rabbit ID), were performed using the estimated marginal means across all groups in R with the emmeans package, and pairwise comparisons were then performed for all pairs using the *t* test with Holm’s adjustment. To compare the slopes from the linear mixed-effects model [log_10_ CFU/mL ~ day + (0 + day|rabbit ID)], coefficients for each rabbit were extracted and analyzed by ANOVA and then Tukey’s honestly significant difference test.

The data for fungal burdens in the mouse lung and brain tissues and the rabbit brain tissue were analyzed using a Kruskal-Wallis test followed by pairwise comparisons using a Wilcoxon rank sum test with Holm’s adjustment (R package rstatix version 0.7.0) (35). The linear and linear mixed-effect models and calculations of the estimated marginal means were constructed in R using the lmerTest (36) and emmeans (version 1.6.3) (37) packages. Figures were made using ggplot2 version 2.3.3.5 (38) and sjPlot (version 2.8.10) (39). The source code will be provided upon request.

The PK/PD of APX2039 were described using a population methodology. A rabbit was considered an “individual” contributing both plasma concentrations and serial estimates of fungal density from the CSF. The PK and PD data were described by the following four inhomogeneous differential equations:

(1) $$XP\left(\text{1}\right)=\;-K_{a}\;\cdot \;X\left(\text{1}\right)$$

(2) $$XP\left(\text{2}\right)\;=\;K_{a}\;\cdot \;X\left(\text{1}\right)\;-\;\left(K_{cp}+\left(\text{SCL/}V\right)\right)\;\cdot \;X\left(\text{2}\right)\;+\;K_{pc}\;\cdot \;X\left(\text{3}\right)$$

(3) $$XP\left(\text{3}\right)\;=\;K_{cp}\;\cdot \;X\left(\text{2}\right)\;-\;K_{pc}\;\cdot \;X\left(\text{3}\right)$$

(4) $$\begin{array}{l} XP\left(\text{4}\right)\;=\;K_{g\text{max}}\;\cdot \;\left(\text{1}-\left(\left(X\left(\text{2}\right)/V\right)\;\cdot \;\cdot \;H_{g}/\left(C_{\text{5}0g}\;\cdot \;\cdot \;H_{g}+\left(X\left(\text{2}\right)/V\right)\;\cdot \;\cdot \;H_{g}\right)\right)\right)\;\cdot \;\left(\text{1}.\text{D}0-\left(X\left(\text{4}\right)/\text{popmax}\right)\right)\;\cdot \;X\left(\text{4}\right)-\\ kk_{\text{max}}\;\cdot \;\left(X\left(\text{2}\right)/V\right)\;\cdot \;\cdot \;H_{k}/\left(C_{50k}\;\cdot \;\cdot \;H_{k}+\left(X\left(\text{2}\right)/V\right)\;\cdot \;\cdot \;H_{k}\right)\;\cdot \;X\left(\text{4}\right) \end{array}$$

where XP is the rate of change with parentheses showing the relevant compartment. For example, XP(1) is the rate of change of drug amount in compartment 1. *K_a_* is the first-order transfer rate constant connecting the gut with the plasma; SCL is the first-order clearance of APX2039 from plasma; *V* is the volume of the central compartment; *K_cp_* and *K_pc_* are the first-order intercompartmental rate constants; *K_g_*_max_ and *K_k_*_max_ are the rates of fungal growth and drug-induced killing, respectively, in CSF; *H_g_* and *H_k_* are the slope functions for the effects of the drug on growth and killing, respectively; *C*_50_*_g_* and *C*_50_*_k_* are the plasma concentrations of APX2039 that result in half-maximal effects on growth and killing, respectively; and popmax is the maximum achievable fungal density in the CSF. An initial condition (i.e., the fungal density in the CSF immediately after inoculation) was estimated as a parameter (not shown in the differential equations).

Equation 1 describes the movement of APX2039 from the gut into the plasma, equation 2 describes the rate of change of the mass of APX2039 in the central compartment (i.e., plasma), equation 3 describes the rate of change of the mass of APX2039 in the peripheral compartment, and equation 4 describes the pharmacodynamics of APX2039 and contains terms that describe the capacity-limited fungal growth in CSF, the drug-induced suppression of growth, and drug-induced fungal killing. Two output equations were used, one to describe the plasma concentrations and the other to describe the time course of the fungal density in the CSF. The structural model was fitted to the combined PK and PD data set using the nonparametric population PK program Pmetrics (40). The adequacy of the fitting was assessed by visual inspection of the observed-predicted results and linear regression of the observed-predicted values for plasma concentrations and pharmacodynamics.

The PK/PD population model was used to link drug exposure with the antifungal effect. A number of pharmacodynamic measures were used, and these included (i) the area under the log_10_ CFU per milliliter-time curve for each rabbit and (ii) the rate of decline of log_10_ CFU per milliliter in CSF. The total drug average AUC_0–24_ in the first 168 h was used as an integrative measure of drug exposure.

## References

[1] Rajasingham R, Smith RM, Park BJ, Jarvis JN, Govender NP, Chiller TM, Denning DW, Loyse A, Boulware DR. 2017. Global burden of disease of HIV-associated cryptococcal meningitis: an updated analysis. Lancet Infect Dis 17:873–881. doi:10.1016/S1473-3099(17)30243-8. 28483415 PMC5818156

[2] Park BJ, Wannemuehler KA, Marston BJ, Govender N, Pappas PG, Chiller TM. 2009. Estimation of the current global burden of cryptococcal meningitis among persons living with HIV/AIDS. AIDS 23:525–530. doi:10.1097/QAD.0b013e328322ffac. 19182676

[3] Anonymous. 2018. WHO guidelines approved by the Guidelines Review Committee, guidelines for the diagnosis, prevention and management of cryptococcal disease in HIV-infected adults, adolescents and children: supplement to the 2016 consolidated guidelines on the use of antiretroviral drugs for treating and preventing HIV infection. World Health Organization, Geneva, Switzerland. 30285342

[4] Jarvis JN, Lawrence DS, Meya DB, Kagimu E, Kasibante J, Mpoza E, Rutakingirwa MK, Ssebambulidde K, Tugume L, Rhein J, Boulware DR, Mwandumba HC, Moyo M, Mzinganjira H, Kanyama C, Hosseinipour MC, Chawinga C, Meintjes G, Schutz C, Comins K, Singh A, Muzoora C, Jjunju S, Nuwagira E, Mosepele M, Leeme T, Siamisang K, Ndhlovu CE, Hlupeni A, Mutata C, van Widenfelt E, Chen T, Wang D, Hope W, Boyer-Chammard T, Loyse A, Molloy SF, Youssouf N, Lortholary O, Lalloo DG, Jaffar S, Harrison TS, Ambition Study Group. 2022. Single-dose liposomal amphotericin B treatment for cryptococcal meningitis. N Engl J Med 386:1109–1120. doi:10.1056/NEJMoa2111904. 35320642 PMC7612678

[5] Perfect JR, Dismukes WE, Dromer F, Goldman DL, Graybill JR, Hamill RJ, Harrison TS, Larsen RA, Lortholary O, Nguyen M-H, Pappas PG, Powderly WG, Singh N, Sobel JD, Sorrell TC. 2010. Clinical practice guidelines for the management of cryptococcal disease: 2010 update by the Infectious Diseases Society of America. Clin Infect Dis 50:291–322. doi:10.1086/649858. 20047480 PMC5826644

[6] Shaw KJ, Ibrahim AS. 2020. Fosmanogepix: a review of the first-in-class broad spectrum agent for the treatment of invasive fungal infections. J Fungi (Basel) 6:239. doi:10.3390/jof6040239. 33105672 PMC7711534

[7] Watanabe NA, Miyazaki M, Horii T, Sagane K, Tsukahara K, Hata K. 2012. E1210, a new broad-spectrum antifungal, suppresses Candida albicans hyphal growth through inhibition of glycosylphosphatidylinositol biosynthesis. Antimicrob Agents Chemother 56:960–971. doi:10.1128/AAC.00731-11. 22143530 PMC3264227

[8] Shaw KJ, Schell WA, Covel J, Duboc G, Giamberardino C, Kapoor M, Moloney M, Soltow QA, Tenor JL, Toffaletti DL, Trzoss M, Webb P, Perfect JR. 2018. In vitro and in vivo evaluation of APX001A/APX001 and other Gwt1 inhibitors against Cryptococcus. Antimicrob Agents Chemother 62:e00523-18. doi:10.1128/AAC.00523-18. 29891599 PMC6105804

[9] Perfect JR, Lang SD, Durack DT. 1980. Chronic cryptococcal meningitis: a new experimental model in rabbits. Am J Pathol 101:177–194. 7004196 PMC1903580

[10] Perfect JR, Durack DT. 1982. Treatment of experimental cryptococcal meningitis with amphotericin B, 5-fluorocytosine, and ketoconazole. J Infect Dis 146:429–435. doi:10.1093/infdis/146.3.429. 6286799

[11] Perfect JR, Durack DT. 1985. Comparison of amphotericin B and *N*-d-ornithyl amphotericin B methyl ester in experimental cryptococcal meningitis and *Candida albicans* endocarditis with pyelonephritis. Antimicrob Agents Chemother 28:751–755. doi:10.1128/AAC.28.6.751. 4083860 PMC180322

[12] Brouwer AE, Rajanuwong A, Chierakul W, Griffin GE, Larsen RA, White NJ, Harrison TS. 2004. Combination antifungal therapies for HIV-associated cryptococcal meningitis: a randomised trial. Lancet 363:1764–1767. doi:10.1016/S0140-6736(04)16301-0. 15172774

[13] Day JN, Chau TT, Lalloo DG. 2013. Combination antifungal therapy for cryptococcal meningitis. N Engl J Med 368:2522–2523. doi:10.1056/NEJMc1305981. 23802521

[14] Molloy SF, Kanyama C, Heyderman RS, Loyse A, Kouanfack C, Chanda D, Mfinanga S, Temfack E, Lakhi S, Lesikari S, Chan AK, Stone N, Kalata N, Karunaharan N, Gaskell K, Peirse M, Ellis J, Chawinga C, Lontsi S, Ndong J-G, Bright P, Lupiya D, Chen T, Bradley J, Adams J, van der Horst C, van Oosterhout JJ, Sini V, Mapoure YN, Mwaba P, Bicanic T, Lalloo DG, Wang D, Hosseinipour MC, Lortholary O, Jaffar S, Harrison TS, ACTA Trial Study Team. 2018. Antifungal combinations for treatment of cryptococcal meningitis in Africa. N Engl J Med 378:1004–1017. doi:10.1056/NEJMoa1710922. 29539274

[15] Dyal J, Akampurira A, Rhein J, Morawski BM, Kiggundu R, Nabeta HW, Musubire AK, Bahr NC, Williams DA, Bicanic T, Larsen RA, Meya DB, Boulware DR, on behalf of the ASTRO-CM Trial Team. 2016. Reproducibility of CSF quantitative culture methods for estimating rate of clearance in cryptococcal meningitis. Med Mycol 54:361–369. doi:10.1093/mmy/myv104. 26768372 PMC4834857

[16] Spickard A, Butler WT, Andriole V, Utz JP. 1963. The improved prognosis of cryptococcal meningitis with amphotericin B therapy. Ann Intern Med 58:66–83. doi:10.7326/0003-4819-58-1-66. 13990086

[17] Utz JP, Garriques IL, Sande MA, Warner JF, Mandell GL, McGehee RF, Duma RJ, Shadomy S. 1975. Therapy of cryptococcosis with a combination of flucytosine and amphotericin B. J Infect Dis 132:368–373. doi:10.1093/infdis/132.4.368. 1185007

[18] Hope W, Stone NRH, Johnson A, McEntee L, Farrington N, Santoro-Castelazo A, Liu X, Lucaci A, Hughes M, Oliver JD, Giamberardino C, Mfinanga S, Harrison TS, Perfect JR, Bicanic T. 2019. Fluconazole monotherapy is a suboptimal option for initial treatment of cryptococcal meningitis because of emergence of resistance. mBio 10:e02575-19. doi:10.1128/mBio.02575-19. 31796539 PMC6890991

[19] van der Horst CM, Saag MS, Cloud GA, Hamill RJ, Graybill JR, Sobel JD, Johnson PC, Tuazon CU, Kerkering T, Moskovitz BL, Powderly WG, Dismukes WE. 1997. Treatment of cryptococcal meningitis associated with the acquired immunodeficiency syndrome. National Institute of Allergy and Infectious Diseases Mycoses Study Group and AIDS Clinical Trials Group. N Engl J Med 337:15–21. doi:10.1056/NEJM199707033370103. 9203426

[20] Katende A, Mbwanji G, Faini D, Nyuri A, Kalinjuma AV, Mnzava D, Hullsiek KH, Rhein J, Weisser M, Meya DB, Boulware DR, Letang E, KIULARCO Study Group. 2019. Short-course amphotericin B in addition to sertraline and fluconazole for treatment of HIV-associated cryptococcal meningitis in rural Tanzania. Mycoses 62:1127–1132. doi:10.1111/myc.12995. 31461550 PMC6834866

[21] Rhein J, Huppler Hullsiek K, Tugume L, Nuwagira E, Mpoza E, Evans EE, Kiggundu R, Pastick KA, Ssebambulidde K, Akampurira A, Williams DA, Bangdiwala AS, Abassi M, Musubire AK, Nicol MR, Muzoora C, Meya DB, Boulware DR, ASTRO-CM Team. 2019. Adjunctive sertraline for HIV-associated cryptococcal meningitis: a randomised, placebo-controlled, double-blind phase 3 trial. Lancet Infect Dis 19:843–851. doi:10.1016/S1473-3099(19)30127-6. 31345462 PMC7041360

[22] Ngan NTT, Mai NTH, Tung NLN, Lan NPH, Tai LTH, Phu NH, Chau NVV, Binh TQ, Hung LQ, Beardsley J, White N, Lalloo D, Krysan D, Hope W, Geskus R, Wolbers M, Nhat LTH, Thwaites G, Kestelyn E, Day J. 2019. A randomized open label trial of tamoxifen combined with amphotericin B and fluconazole for cryptococcal meningitis. Wellcome Open Res 4:8. doi:10.12688/wellcomeopenres.15010.1. 30801037 PMC6381443

[23] Bicanic T, Muzoora C, Brouwer AE, Meintjes G, Longley N, Taseera K, Rebe K, Loyse A, Jarvis J, Bekker LG, Wood R, Limmathurotsakul D, Chierakul W, Stepniewska K, White NJ, Jaffar S, Harrison TS. 2009. Independent association between rate of clearance of infection and clinical outcome of HIV-associated cryptococcal meningitis: analysis of a combined cohort of 262 patients. Clin Infect Dis 49:702–709. doi:10.1086/604716. 19613840 PMC2965403

[24] Pullen MF, Hullsiek KH, Rhein J, Musubire AK, Tugume L, Nuwagira E, Abassi M, Ssebambulidde K, Mpoza E, Kiggundu R, Akampurira A, Nabeta HW, Schutz C, Evans EE, Rajasingham R, Skipper CP, Pastick KA, Williams DA, Morawski BM, Bangdiwala AS, Meintjes G, Muzoora C, Meya DB, Boulware DR. 2020. Cerebrospinal fluid early fungicidal activity as a surrogate endpoint for cryptococcal meningitis survival in clinical trials. Clin Infect Dis 71:e45–e49. doi:10.1093/cid/ciaa016. 31912875 PMC7755087

[25] Perfect JR, Wright KA. 1994. Amphotericin B lipid complex in the treatment of experimental cryptococcal meningitis and disseminated candidosis. J Antimicrob Chemother 33:73–81. doi:10.1093/jac/33.1.73. 8157577

[26] Lestner J, McEntee L, Johnson A, Livermore J, Whalley S, Schwartz J, Perfect JR, Harrison T, Hope W. 2017. Experimental models of short courses of liposomal amphotericin B for induction therapy for cryptococcal meningitis. Antimicrob Agents Chemother 61:e00090-17. doi:10.1128/AAC.00090-17. 28320715 PMC5444125

[27] Ponatshego PL, Lawrence DS, Youssouf N, Molloy SF, Alufandika M, Bango F, Boulware DR, Chawinga C, Dziwani E, Gondwe E, Hlupeni A, Hosseinipour MC, Kanyama C, Meya DB, Mosepele M, Muthoga C, Muzoora CK, Mwandumba H, Ndhlovu CE, Rajasingham R, Sayed S, Shamu S, Tsholo K, Tugume L, Williams D, Maheswaran H, Shiri T, Boyer-Chammard T, Loyse A, Chen T, Wang D, Lortholary O, Lalloo DG, Meintjes G, Jaffar S, Harrison TS, Jarvis JN, Niessen LW. 2019. AMBIsome Therapy Induction OptimisatioN (AMBITION): high dose AmBisome for cryptococcal meningitis induction therapy in sub-Saharan Africa: economic evaluation protocol for a randomised controlled trial-based equivalence study. BMJ Open 9:e026288. doi:10.1136/bmjopen-2018-026288. PMC6500286 30940760

[28] Perfect JR, Savani DV, Durack DT. 1986. Comparison of itraconazole and fluconazole in treatment of cryptococcal meningitis and candida pyelonephritis in rabbits. Antimicrob Agents Chemother 29:579–583. doi:10.1128/AAC.29.4.579. 3010846 PMC180445

[29] Perfect JR, Wright KA, Hobbs MM, Durack DT. 1989. Treatment of experimental cryptococcal meningitis and disseminated candidiasis with SCH39304. Antimicrob Agents Chemother 33:1735–1740. doi:10.1128/AAC.33.10.1735. 2556078 PMC172747

[30] Kovanda LL, Giamberardino C, McEntee L, Toffaletti DL, Franke KS, Bartuska A, Smilnak G, de Castro GC, Ripple K, Hope WW, Perfect JR. 2019. Pharmacodynamics of isavuconazole in a rabbit model of cryptococcal meningoencephalitis. Antimicrob Agents Chemother 63:e00546-19. doi:10.1128/AAC.00546-19. 31209006 PMC6709487

[31] Livermore J, Howard SJ, Sharp AD, Goodwin J, Gregson L, Felton T, Schwartz JA, Walker C, Moser B, Müller W, Harrison TS, Perfect JR, Hope WW. 2014. Efficacy of an abbreviated induction regimen of amphotericin B deoxycholate for cryptococcal meningoencephalitis: 3 days of therapy is equivalent to 14 days. mBio 5:e00725-13. doi:10.1128/mBio.00725-13. 24473125 PMC3903272

[32] Wickham H, Averick M, Bryan J, Chang W, D’Agostino McGowan L, François R, Grolemund G, Hayes A, Henry L, Hester J, Kuhn M, Pedersen TL, Miller E, Bache SM, Müller K, Ooms J, Robinson D, Seidel DP, Spinu V, Takahashi K, Vaughan D, Wilke C, Woo K, Yutani H. 2019. Welcome to the tidyverse. J Open Source Softw 4:1686. doi:10.21105/joss.01686.

[33] Wickham H. 2019. stringr: simple, consistent wrappers for common string operations.

[34] R Core Team. 2021. R: a language and environment for statistical computing. R Foundation for Statistical Computing, Vienna, Austria. https://www.R-project.org/.

[35] Kassambara A. 2021. rstatix: pipe-friendly framework for basic statistical tests.

[36] Kuznetsova A, Brockhoff PB, Christensen RHB. 2017. lmerTest package: tests in linear mixed effects models. J Stat Softw 82:1–26. doi:10.18637/jss.v082.i13.

[37] Lenth RV. 2021. emmeans: estimated marginal means, aka least-squares means.

[38] Wickham H. 2016. ggplot2: elegant graphics for data analysis. Springer-Verlag, New York, NY.

[39] Lüdecke D. 2021. sjPlot: data visualization for statistics in social science.

[40] Neely MN, van Guilder MG, Yamada WM, Schumitzky A, Jelliffe RW. 2012. Accurate detection of outliers and subpopulations with Pmetrics, a nonparametric and parametric pharmacometric modeling and simulation package for R. Ther Drug Monit 34:467–476. doi:10.1097/FTD.0b013e31825c4ba6. 22722776 PMC3394880

