# Supplementary material for: Efficacy of APX2039 in a Rabbit Model of Cryptococcal Meningitis
Source: mBio. 2022 Oct 12;13(6):e02347-22. doi: 10.1128/mbio.02347-22 (PMC9765414; doi:10.1128/mbio.02347-22)
Supplement: TABLE S1 [file mbio.02347-22-s0001.docx]

| Supplemental Table 1: Pairwise Comparisons for the reduction in log_10_ CFU/mL of CSF | | | | | | |
| --- | --- | --- | --- | --- | --- | --- |
| group1 | group2 | p | p.adj | p.format | p.signif | method |
| APX2039 (50 mg/kg, QD) | APX2039 (75 mg/kg, QD) | 0.285714286 | 1 | 0.28571 | ns | Wilcoxon |
| APX2039 (50 mg/kg, QD) | APX2039 (50 mg/kg, BID) | 0.004040404 | 0.081 | 0.00404 | ** | Wilcoxon |
| APX2039 (50 mg/kg, QD) | Amphotericin B | 0.015873016 | 0.24 | 0.01587 | * | Wilcoxon |
| APX2039 (50 mg/kg, QD) | APX2039 (25 mg/kg, QD) | 0.412698413 | 1 | 0.4127 | ns | Wilcoxon |
| APX2039 (50 mg/kg, QD) | Fosmanogepix | 0.476190476 | 1 | 0.47619 | ns | Wilcoxon |
| APX2039 (50 mg/kg, QD) | Fluconazole | 0.063492063 | 0.6 | 0.06349 | ns | Wilcoxon |
| APX2039 (50 mg/kg, QD) | Control | 0.05034965 | 0.6 | 0.05035 | ns | Wilcoxon |
| APX2039 (75 mg/kg, QD) | APX2039 (50 mg/kg, BID) | 0.040149155 | 0.52 | 0.04015 | * | Wilcoxon |
| APX2039 (75 mg/kg, QD) | Amphotericin B | 0.547619048 | 1 | 0.54762 | ns | Wilcoxon |
| APX2039 (75 mg/kg, QD) | APX2039 (25 mg/kg, QD) | 0.055555556 | 0.6 | 0.05556 | ns | Wilcoxon |
| APX2039 (75 mg/kg, QD) | Fosmanogepix | 0.051948052 | 0.6 | 0.05195 | ns | Wilcoxon |
| APX2039 (75 mg/kg, QD) | Fluconazole | 0.69047619 | 1 | 0.69048 | ns | Wilcoxon |
| APX2039 (75 mg/kg, QD) | Control | 0.000999001 | 0.026 | 0.001 | *** | Wilcoxon |
| APX2039 (50 mg/kg, BID) | Amphotericin B | 0.065268065 | 0.6 | 0.06527 | ns | Wilcoxon |
| APX2039 (50 mg/kg, BID) | APX2039 (25 mg/kg, QD) | 0.001554002 | 0.036 | 0.00155 | ** | Wilcoxon |
| APX2039 (50 mg/kg, BID) | Fosmanogepix | 0.000666001 | 0.018 | 0.00067 | *** | Wilcoxon |
| APX2039 (50 mg/kg, BID) | Fluconazole | 0.001554002 | 0.036 | 0.00155 | ** | Wilcoxon |
| APX2039 (50 mg/kg, BID) | Control | 8.23E-05 | 0.0023 | 8.20E-05 | **** | Wilcoxon |
| Amphotericin B | APX2039 (25 mg/kg, QD) | 0.007936508 | 0.13 | 0.00794 | ** | Wilcoxon |
| Amphotericin B | Fosmanogepix | 0.004329004 | 0.082 | 0.00433 | ** | Wilcoxon |
| Amphotericin B | Fluconazole | 0.007936508 | 0.13 | 0.00794 | ** | Wilcoxon |
| Amphotericin B | Control | 0.000999001 | 0.026 | 0.001 | *** | Wilcoxon |
| APX2039 (25 mg/kg, QD) | Fosmanogepix | 0.930735931 | 1 | 0.93074 | ns | Wilcoxon |
| APX2039 (25 mg/kg, QD) | Fluconazole | 0.015873016 | 0.24 | 0.01587 | * | Wilcoxon |
| APX2039 (25 mg/kg, QD) | Control | 0.146853147 | 1 | 0.14685 | ns | Wilcoxon |
| Fosmanogepix | Fluconazole | 0.004329004 | 0.082 | 0.00433 | ** | Wilcoxon |
| Fosmanogepix | Control | 0.002797203 | 0.059 | 0.0028 | ** | Wilcoxon |
| Fluconazole | Control | 0.000999001 | 0.026 | 0.001 | *** | Wilcoxon |
