# Supplementary figures and images for: Efficacy of APX2039 in a Rabbit Model of Cryptococcal Meningitis
Source: mBio. 2022 Oct 12;13(6):e02347-22. doi: 10.1128/mbio.02347-22 (PMC9765414; doi:10.1128/mbio.02347-22)

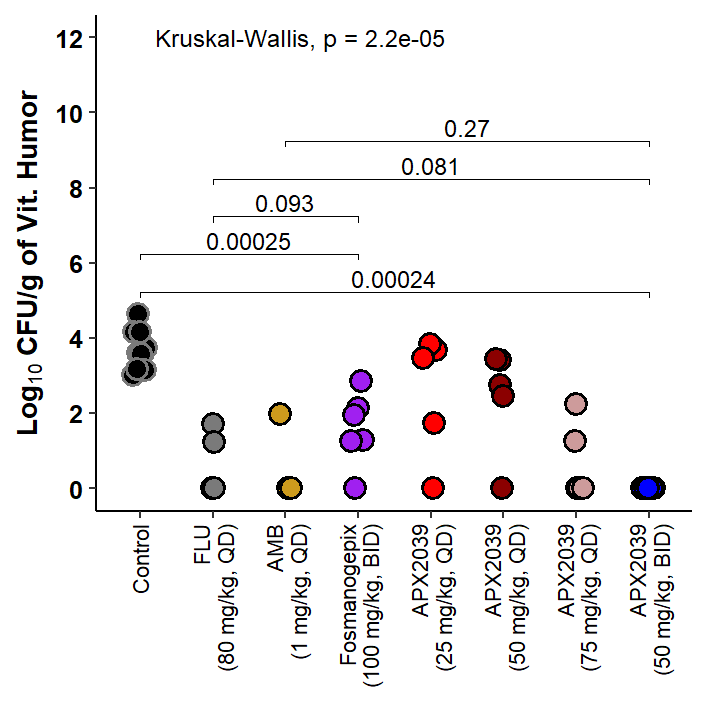

Supplement: FIG S1 [file mbio.02347-22-s0002.tif]
